# Effects of Treatment With Cyclosporine Ophthalmic Solution 0.09% on Dry Eye Questionnaire Scores Among Patients With Dry Eye Disease

**DOI:** 10.7759/cureus.110141

**Published:** 2026-06-02

**Authors:** Rana Taji, King Y Chow, Shazia Hassan, Belinda Yap

**Affiliations:** 1 Optometry, Toronto Medical Eye Associates, North York, CAN; 2 Ophthalmology, Clarity Eye Institute, Newmarket, CAN; 3 Biostatistics, Cencora, Innomar Strategies Inc., Oakville, CAN

**Keywords:** cequa, dry eye questionnaire, keratoconjunctivitis sicca, long-term treatment, retrospective study

## Abstract

Objective

This multicenter, retrospective study was conducted to evaluate patient-reported scores for the 5-part Dry Eye Questionnaire (DEQ-5) over a six-month treatment period using cyclosporine ophthalmic solution 0.09% (CsA 0.09%).

Materials and methods

Patients with moderate-to-severe dry eye disease (DED) who completed the DEQ-5 at baseline and at least one post-baseline assessment after one, three, and six months of twice-daily CsA 0.09% treatment were included in the study. Adverse events were recorded.

Results

A total of 392 patients entered the study. At baseline, the mean (standard deviation (SD)) DEQ-5 score was 14.7 (3.4). The scores improved to 12.6 (4.1), 11.9 (4.2), and 10.8 (4.4) at one, three, and six months, respectively, corresponding with reduced DED symptoms. Most patients reported improvements in DEQ-5 scores at each time point; by month six, 69/88 (78.4%) patients reported improved DEQ-5 scores from baseline, while 8/88 (9.1%) reported no changes and 11/88 (12.5%) reported progression of DED symptoms.

Conclusions

CsA 0.09% was well tolerated and led to significant improvements in DEQ-5 scores, reflecting better management of DED symptoms at one, three, and six months of treatment. These results highlight the potential of CsA 0.09% as a long-term treatment option for moderate to severe DED.

## Introduction

Dry eye disease (DED) is a multifactorial, chronic ocular surface disorder characterized by reduced tear production and/or disruption of tear film homeostasis, which triggers a cycle of ocular surface inflammation and damage that results in symptoms such as ocular burning, stinging, grittiness, and/or discomfort [1-3]. To interrupt this cycle of inflammation, many DED treatments target the underlying inflammatory component of DED using drugs such as the calcineurin inhibitor and immunomodulatory agent cyclosporine A [2]. Multiple formulations of cyclosporine A are approved by the U.S. Food and Drug Administration to treat DED and are indicated to increase tear production, including cyclosporine ophthalmic solution 0.09% (CsA 0.09%; CEQUA®), 0.1% (Vevye®), and cyclosporine ophthalmic emulsion 0.05% (Restasis®) [4-7]. Due to the hydrophobic nature of cyclosporine A, some DED treatments rely on emulsions to enable its use in ophthalmic formulations. Alternatively, CsA 0.09% uses a nanomicellar formulation to encapsulate cyclosporine A and enhance its interaction with the ocular surface, which may reduce side effects associated with ophthalmic emulsions, such as instillation site pain [8].

Clinical studies support the safety and efficacy of CsA 0.09% for the treatment of DED signs and symptoms after 12 weeks of treatment [9,10]. However, few studies have evaluated patient-reported symptoms of DED during long-term treatment with CsA 0.09%. Patient-reported outcomes are essential for the diagnosis and management of DED, as they provide insights into symptom burden and quality of life [11]. The validated 5-part Dry Eye Questionnaire (DEQ-5) is a commonly used instrument that measures DED symptoms using a 5 or 6-point scale for each item and offers insight into patient quality of life [12]. Therefore, this study was conducted to evaluate changes in patient-reported DEQ-5 scores and to monitor safety at one, three, and six months of CsA 0.09% treatment in patients with moderate-to-severe DED.

## Materials and methods

Study design and oversight

This multicenter retrospective study was conducted across five clinics in Ontario, Canada, between March 2022 and April 2024. All included patients provided consent for retrospective data collection from medical records obtained during routine clinical visits. The study was approved by an institutional review board (Veritas IRB Inc., overall study number: 2025-3444-20782-1; individual site numbers: 2025-3444-20783-1, 2025-3444-20784-1, 2025-3444-20785-1, and 2025-3444-20786-1) and was deemed exempt because all interventions in the protocol were part of standard clinical practice.

Study population

**Figure 1:**
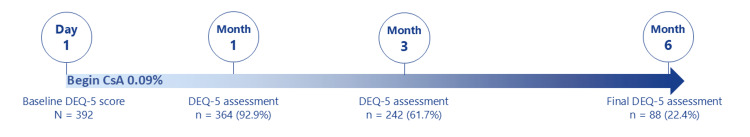
Study design CsA 0.09%: cyclosporine ophthalmic solution 0.09%; DEQ-5: 5-part Dry Eye Questionnaire Image credit: the authors

Patients 18 years of age or older with a clinically confirmed diagnosis of DED were included in the study. Only patients with moderate or severe DED symptoms (defined as a baseline score greater than 6 on the DEQ-5) were included. Patients were required to complete a baseline DEQ-5 assessment as well as at least one follow-up assessment at one, three, or six months during treatment to be included in the analysis. All patients were treated with one drop in each eye twice daily for up to six months (Figure 1). Any patients with a history of intolerance to topical cyclosporine were excluded from the study.

Endpoint measures

The primary endpoint was the mean change from baseline in the DEQ-5 scores at one, three, and six months. The one-month time point included patients with a follow-up visit within 60 days of baseline. For the three and six-month time points, DEQ-5 scores were collected from patients who attended follow-up visits at three and six months (± 30 days). Safety was assessed by collecting and reporting adverse events. The DEQ-5 asked patients to score their DED symptoms on a 5- or 6-point scale (depending on the question), as follows [12]: (1) During a typical day in the past month, how often did your eyes feel discomfort (scored 0-4)? (2) When your eyes felt discomfort, how intense was this feeling of discomfort at the end of the day, within two hours of going to bed (scored 0-5)? (3) During a typical day in the past month, how often did your eyes feel dry (scored 0-4)? (4) When your eyes felt dry, how intense was this feeling of dryness at the end of the day, within two hours of going to bed (scored 0-5)? (5) During a typical day in the past month, how often did your eyes look or feel excessively watery (scored 0-4)?

Points from each question were summed up to give a total score representative of the patients’ DED symptoms (referred to as “DEQ-5 score”). Higher DEQ-5 scores indicate more severe DED symptoms, whereas lower scores indicate milder symptoms; the maximum possible DEQ-5 score is 22 [12].

Statistical analysis

Continuous variables were reported using frequency, mean, and standard deviation (SD). Categorical variables were reported as counts and percentages; outcome measures were estimated at six months (± 2 months). Baseline DEQ-5 scores were grouped into moderate (7 to 12) and severe (≥12) categories. Mean changes from baseline DEQ-5 scores were analyzed using a paired t-test at each post-baseline time point (one, three, and six months). Missing data were not imputed; only patients with available post-baseline DEQ-5 scores at each time point were included in that specific analysis. Patients who missed a post-baseline DEQ-5 assessment were included in later time points if they remained on CsA 0.09% throughout the study period. Patient symptoms were also classified as improved, no change, or progressed based on changes in the DEQ-5 scores from baseline.

## Results

Patient demographics and clinical characteristics

A total of 392 patients with moderate or severe DED were enrolled (Table 1). The mean (SD) age was 61.4 (14.7) years, and most patients were female (n = 311, 79.3%). Patients had a mean (SD) duration of DED of 2.5 (3.6) years. The overall mean (SD) baseline DEQ-5 score was 14.7 (3.4); the mean (SD) DEQ-5 score for patients with moderate DED (n = 106, 27.0%) and severe DED (n = 286, 73.0%) at baseline was 10.3 (1.5) and 16.4 (2.4), respectively.

**Table 1:** Patient demographics and baseline characteristics ^a^Patients with mild DEQ-5 scores at baseline (<6 points) were not eligible for the study Notes: Data are presented for all patients with a baseline DEQ-5 score and at least one post-baseline follow-up DED: dry eye disease; DEQ-5: 5-part Dry Eye Questionnaire; SD: standard deviation

Variables	Values (N = 392)
Age, years, mean (SD)	61.4 (14.7)
Duration of DED, years, mean (SD)	2.5 (3.6)
Sex, n (%)	
Female	311 (79.3)
Male	81 (20.7)
Patients in each DEQ-5 category^a^, n (%)	
7–12 (moderate)	106 (27.0)
>12 (severe)	286 (73.0)
Baseline DEQ-5 score^a^, mean (SD)	
7–12 (moderate)	10.3 (1.5)
>12 (severe)	16.4 (3.4)
All patients	14.7 (3.4)

DEQ-5 scores

**Figure 2:**
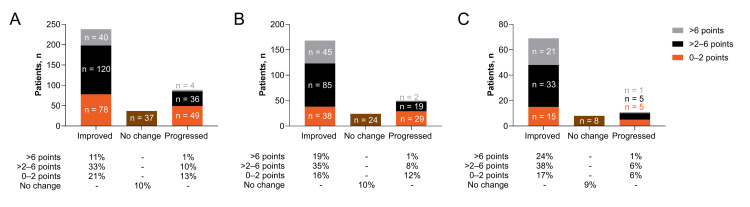
Change from baseline in DEQ-5 scores at (A) one, (B) three, and (C) six months DEQ-5: 5-part Dry Eye Questionnaire

Of the 364/392 (92.9%) patients who had a baseline and one-month follow-up visit, 238/364 (65.4%) reported a significant improvement in mean (SD) DEQ-5 scores from 14.7 (3.4) at baseline to 12.6 (4.1) at one month (*p* < 0.001; Figure 2A, Table 2). Among these patients who showed improvement, 78/238 (32.8%) patients had an improvement from baseline DEQ-5 scores between >0 and 2 points, 120/238 (50.4%) patients improved by >2 to 6 points, and 40/238 (16.8%) patients improved by >6 points. Thirty-seven of 364 (10.2%) patients had no change in DEQ-5 score from baseline, and 89/364 (24.5%) reported an increase in DEQ-5 scores from baseline.

**Table 2:** Summary of mean DEQ-5 scores DEQ-5: 5-part Dry Eye Questionnaire; SD: standard deviation

Visit	Patients, n (%)	DEQ-5 score, mean (SD)	*P*-value (vs. baseline)
Baseline	392 (100)	14.7 (3.4)	
1 month	364 (92.9)	12.6 (4.1)	< 0.001
3 months	242 (61.7)	11.9 (4.2)	< 0.001
6 months	88 (22.4)	10.8 (4.4)	< 0.001

Among patients who had a baseline and three-month follow-up visit (n = 242), 168/242 (69.4%) reported a significant improvement in mean (SD) DEQ-5 scores from 14.7 (3.4) at baseline to 11.9 (4.2) at three months (*p* < 0.001; Figure 2B, Table 2). Among the 168 patients who showed improvement, 38/168 (22.6%) patients had an improvement from baseline DEQ-5 scores between >0 and 2, 85/168 (50.6%) patients improved by >2 to 6 points, and 45/168 (26.8%) patients improved by >6 points. Twenty-four of 242 (9.9%) patients had no change in DEQ-5 scores, and 50/242 (20.7%) had an increase in DEQ-5 scores and worsening of DED symptoms from baseline.

A total of 88 patients had a baseline and six-month follow-up visit, and 69/88 (78.4%) patients reported a significant improvement in mean (SD) DEQ-5 scores from 14.7 (3.4) at baseline to 10.8 (4.4) at six months (*p* < 0.001; Figure 2C, Table 2). Among the 69 patients who showed improvement, 15/69 (21.7%) patients had an improvement from baseline DEQ-5 scores between >0 and 2, 33/69 (47.8%) patients improved by >2 to 6 points, and 21/69 (30.4%) patients improved by >6 points. Eight of 88 (9.1%) patients had no change in DEQ-5 scores, and 11/88 (12.5%) had an increase in DEQ-5 scores and progression of DED symptoms from baseline.

**Figure 3:**
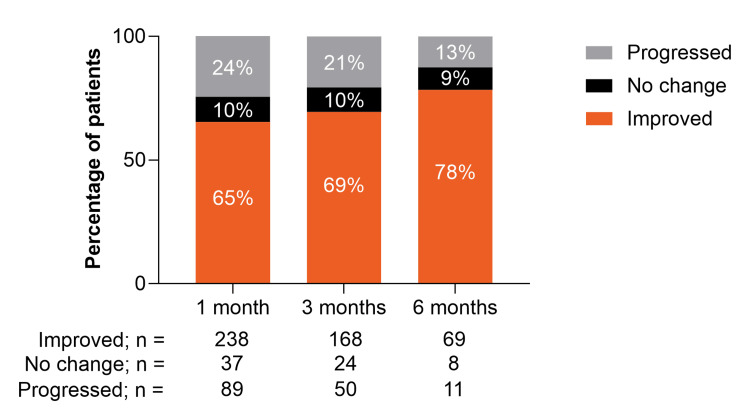
Summary of patient outcomes

Across the three time points assessed, most patients demonstrated a significant improvement from baseline (*p* <0.001) in DEQ-5 scores following treatment with twice-daily CsA 0.09% (Figure 3).

Safety

Overall, 157 treatment-emergent adverse events (TEAEs) were reported by 122/392 (31.1%) patients during the treatment period (Table 3). The most frequently reported ocular TEAEs (>5% of patients) were instillation-site pain (96/157, 61.1%), eye irritation (25/157, 15.9%), conjunctival hyperemia (10/157, 6.4%), and eye pruritus (8/157, 5.1%); the non-ocular TEAEs reported by patients were headache (3/157, 1.9%), urinary tract infection (2/157, 1.3%), and sinusitis (2/157, 1.3%). Overall, 56/392 (14.3%) patients discontinued treatment or were lost to follow-up.

**Table 3:** Overview of TEAEs TEAE: treatment-emergent adverse event

Variables	Values (N = 392)
Patients with ≥ 1 TEAE, n (%)	122 (31.1)
Total number of TEAEs	157
Ocular TEAEs most frequently reported, events (%)	
Instillation-site pain	96 (61.1)
Eye irritation	25 (15.9)
Conjunctival hyperemia	10 (6.4)
Eye pruritus	8 (5.1)
All non-ocular TEAEs reported, events (%)	
Headache	3 (1.9)
Urinary tract infection	2 (1.3)
Sinusitis	2 (1.3)
Patients with TEAEs leading to study drug discontinuation or lost to follow-up, n (%)	56 (14.3)

## Discussion

This retrospective study is the first to assess changes in patient-reported DED symptoms with CsA 0.09% treatment for up to six months. Patients treated with twice-daily CsA 0.09% experienced significant improvements from baseline in reported DEQ-5 scores, beginning at the first post-baseline time point (one month) and continuing through the end of the study. Mean (SD) DEQ-5 scores significantly decreased from a baseline score of 14.7 (3.4) to 12.6 (4.1), 11.9 (4.2), and 10.8 (4.4) at one, three, and six months, respectively, corresponding to reduced DED symptoms and improved quality of life in patients with DED. In this study, most patients (>65%) reported improvements from baseline in DEQ-5 scores at every time point (one month: 65.4%, 238/364; 3 months: 69.4%, 168/242), increasing to 78% (69/88) after six months of treatment. CsA 0.09% was well tolerated in this patient population, consistent with its safety profile in the Phase 2b/3, Phase 3, and Phase 4 clinical studies [9,10,13-15]. Overall, 122/392 (31.1%) patients reported TEAEs, and the most frequently reported ocular TEAEs were instillation-site pain (96/157, 61.1%) and eye irritation (25/157, 15.9%).

Questionnaires like the DEQ-5 are an important clinical tool to quickly evaluate the patient’s perceived symptom severity and disease burden, and they may help guide treatment decisions [11]. The Ocular Surface Disease Index (OSDI) is another frequently used questionnaire in clinical studies, and it assesses patient-reported DED symptom severity using a 12-question test that asks patients to score their symptoms on a 0- to 4-point scale and calculates a total score out of 100 points [16]. A recent meta-analysis of 21 randomized clinical studies that compared different CsA formulations for the treatment of DED found that CsA ophthalmic emulsion 0.05% (Restasis®) demonstrated significant improvements in OSDI scores vs. placebo (odds ratio: −4.82; 95% CI: −6.18 to −3.45) and was ranked the most effective DED treatment based on patient-reported scores by the surface under cumulative ranking method [17]. Importantly, CsA 0.09% was not ranked in patient-reported outcomes in this meta-analysis because the Phase 2b/3 and Phase 3 clinical studies with CsA 0.09% used a two-question modified Symptom Assessment iN Dry Eye (mSANDE) questionnaire instead of the OSDI and reported the numbers of patients who improved rather than questionnaire scores [17].

The Phase 2b/3 and Phase 3 studies reported approximately a mean 30% improvement from baseline mSANDE scores in patients who received both placebo and CsA 0.09% (not statistically significant vs. placebo) during the 84-day treatment period [9,10]. However, in a Phase 4 study, patients whose DED symptoms were inadequately controlled (still symptomatic for three months or longer) on CsA ophthalmic emulsion 0.05% who switched immediately to CsA 0.09% reported statistically significant improvements in mSANDE scores as early as the first post-baseline follow-up (Week 4), with these improvements continuing throughout the 12-week treatment period [13]. While there are many questionnaires used by clinicians to quantify aspects of disease management, we chose the validated DEQ-5 for its brevity, reliability, and focus on DED symptoms, as longer questionnaires may introduce patient fatigue or may be designed for broader use and detection of multiple ocular surface diseases [11,12].

Certain limitations should be taken into account while interpreting the results of this study. First, while objective tests and patient questionnaires are both critical to monitoring DED management [11], patient-reported scores alone may not always correlate with improvements in the clinical signs of DED. Also, the open-label nature of this retrospective study may bias patients towards preferring the study drug and influence DEQ-5 scores. Notably, while more patients did experience improvements in DED symptoms at each time point, the increase in the percentage of patients reporting an improvement may be overestimated if patients who experienced no change or progression of DED symptoms discontinued study treatment. In the six-month study period, 122/392 (31.1%) patients experienced adverse events, and 56/392 (14.3%) discontinued treatment due to TEAEs or were lost to follow-up, so later time points will be needed in future studies to fully assess the long-term tolerability of CsA 0.09% treatment.

However, particularly at later time points, it is important to note that there was still a significant decrease from baseline (*p* < 0.001) in DEQ-5 scores at the earliest time point (one month), corresponding to improved DED symptom management. In addition, while mean DEQ-5 scores improved at each time point compared with baseline, it is important to note that scores overall remained in the moderate-to-severe range for DEQ-5, which underscores the chronic, recalcitrant nature of DED. Lastly, demographic data were not collected during this study, which may affect the generalizability of the conclusions of the study.

Despite these limitations, these results support the long-term use of CsA 0.09% in patients with DED. The DEQ-5 may be used to rapidly assess patient DED symptoms, and treatment satisfaction remains an important aspect of DED management.

## Conclusions

Overall, twice-daily CsA 0.09% therapy resulted in significantly more patients reporting improvements from baseline in DED symptoms as assessed by DEQ-5 scores at one, three, and six months of treatment. CsA 0.09% was well tolerated, and the adverse events observed were similar to those reported in previous clinical studies. These results support the potential of CsA 0.09% for the long-term treatment of DED.
